# Association between autism and dementia across generations: evidence from a family study of the Swedish population

**DOI:** 10.1038/s41380-025-03045-6

**Published:** 2025-05-14

**Authors:** Zheng Chang, Honghui Yao, Shihua Sun, Le Zhang, Shengxin Liu, Isabell Brikell, Brian M. D’Onofrio, Henrik Larsson, Paul Lichtenstein, Ralf Kuja-Halkola, Sara Hägg, Francesca Happé, Mark J. Taylor

**Affiliations:** 1 Department of Medical Epidemiology and Biostatistics, Karolinska Institutet, Stockholm, Sweden; 2 Department of Global Public Health and Primary Care, University of Bergen, Bergen, Norway; 3 Department of Biomedicine, University of Bergen, Bergen, Norway; 4 Department of Psychological and Brain Sciences, Indiana University, Bloomington, Indiana USA; 5 School of Medical Sciences, Örebro University, Örebro, Sweden; 6 Social, Developmental, and Genetic Psychiatry Centre, Institute of Psychiatry, Psychology, and Neuroscience, King’s College London, London, UK

**Keywords:** Autism spectrum disorders, Genetics

## Abstract

There is emerging evidence to suggest that autistic individuals are at an increased risk for cognitive decline or dementia. It is unknown whether this association is due to shared familial influences between autism and dementia. The main purpose of this study was, thus, to investigate the risk of dementia in relatives of autistic individuals. We conducted a family study based on linking Swedish registers. We identified all individuals born in Sweden from 1980–2013, followed until 2020, and clinical diagnoses of autism among these individuals. We linked these index individuals with their parents, grandparents, and aunts/uncles. The risk of dementia (including any type of dementia, Alzheimer’s disease, and other types of dementia) in relatives of autistic individuals was estimated using Cox proportional hazards models. Analyses were then stratified by sex of the relatives and intellectual disability in autistic individuals. Relatives of autistic individuals were at an increased risk of dementia. The risk was strongest in parents (hazards ratio [HR] = 1.36, 95% confidence intervals = 1.25–1.49), and weaker in grandparents (HR = 1.08, 1.06–1.10) and aunts/uncles (HR = 1.15, 0.96–1.38). Furthermore, there were indications of a stronger association between autism in index individuals and dementia in mothers (HR = 1.51, 1.29–1.77) compared to dementia in fathers (HR = 1.30, 1.16–1.45). There was only a small difference in relatives of autistic individuals with and without intellectual disability. Our results provide evidence of familial co-aggregation between autism and different types of dementia, and a potential genetic link. Future research now needs to clarify the risk of dementia in autistic individuals.

## Introduction

Autism is a neurodevelopmental condition that affects social communication and behavioral flexibility. While historically conceptualized as a condition of childhood, it is now established that most autistic individuals continue to meet the diagnostic criteria for autism as adults. More recent lines of evidence highlight the presence of diagnoses and traits of autism in populations of age, calling for the need for a lifespan perspective in understanding the needs of autistic people [1]. To date, existing studies show that autistic individuals of age are at an increased risk for depression [2] and certain somatic conditions associated with aging [3], yet there remains scant evidence concerning the other outcomes experienced by autistic individuals as they age [1].

A pertinent age-related condition, which has received little research focus in relation to autism, is dementia. Dementia is characterized by severe cognitive decline, which has considerable impacts on everyday functioning [4]. There are multiple subtypes of dementia, with Alzheimer’s disease being the most common [4]. Existing studies on autism and dementia paint a mixed picture. Researchers have proposed that autism could be a protective factor against dementia, which is partially supported by a Dutch study showing that decline in visual memory was slower in older autistic adults compared to non-autistic individuals [5], while a subsequent study found that there was no difference between autistic and nonautistic individuals in cognitive decline [6]. On the other hand, there is emerging evidence to suggest that autistic individuals are at an increased risk for dementia. One study of medical insurance records in the USA reported that 25.2% of autistic individuals aged over 65 had a cognitive disorder (with dementia included in this category), compared to 4.9% of non-autistic individuals [7]. A study of autistic adults aged 42–81 enrolled in Simons Powering Autism Research reported that up to 48.9% of autistic individuals had cognitive decline, based on a dementia screening tool [8]. In the only study that we are aware of to have reported the risk of dementia in autistic individuals, which was based on medical insurance records in the USA, 4.84% of autistic individuals aged 30–64 had dementia, compared to 0.97% of non-autistic individuals. The mean age of onset was also lower in autistic individuals [9].

There is, thus, a need for more research clarifying the association between autism and dementia. This endeavor is complicated, however, by the lack of available cohorts of autistic individuals of old age. Existing cohorts likely under-sample older autistic adults, because the diagnosis was less widely used when such cohorts were younger. An avenue to address this challenge lies in genetically informative designs, such as cross-generational family studies. In studying whether there are familial links between autism and dementia, it would subsequently be possible to draw predictions concerning the risk of dementia in autistic individuals and ensure that more targeted screening is put in place. The most recent genome-wide association studies (GWAS) of autism and dementia detected 4 and 75 loci associated with each condition respectively. These studies did not report significant genetic correlations between common variants associated with these conditions, however [10, 11]. A recent Mendelian randomization study also did not detect a causal association between genetic factors associated with autism and dementia [12]. However, these studies focused only on common genetic variation, while the autism GWAS was considerably smaller than the dementia GWAS. There is, therefore, considerable value in conducting family studies of autism and dementia as a first step towards understanding their association.

We therefore conducted a family study of the association between autism and dementia. Specifically, we tested whether the parents, grandparents, and aunts or uncles of autistic individuals are at an increased risk for dementia. Given that the male to female ratio in autism diagnoses has been reported to be 3:1 [13], we also examined these associations stratified by sex of autistic individuals. Finally, we also aimed to assess whether the associations differ between autistic individuals with and without a co-occurring intellectual disability.

## Materials and methods

### Data sources

We linked several nationwide Swedish population-based registers using the unique personal identification number assigned to residents of Sweden. These included the Medical Birth Register (MBR), recording over 98% of births in Sweden since 1973 [14]. The National Patient Register (NPR) provides diagnostic data, covering inpatient care since 1973 (with nationwide coverage from 1987) and outpatient care from 2001 [15]. The Multi-Generation Register documents biological relationships for individuals residing in Sweden since 1932 [16]. The Cause of Death Register records of deaths and causes of death based on ICD codes, with full coverage since 1961 [17]. The Prescribed Drug Register records data on all dispensations of prescribed medication in Sweden since July 2005, recording the Anatomical Therapeutic Chemical codes for each medication [18]. The Total Population Register provides demographic information on residents of Sweden, including information on migration.

### Participants

**Fig. 1:**
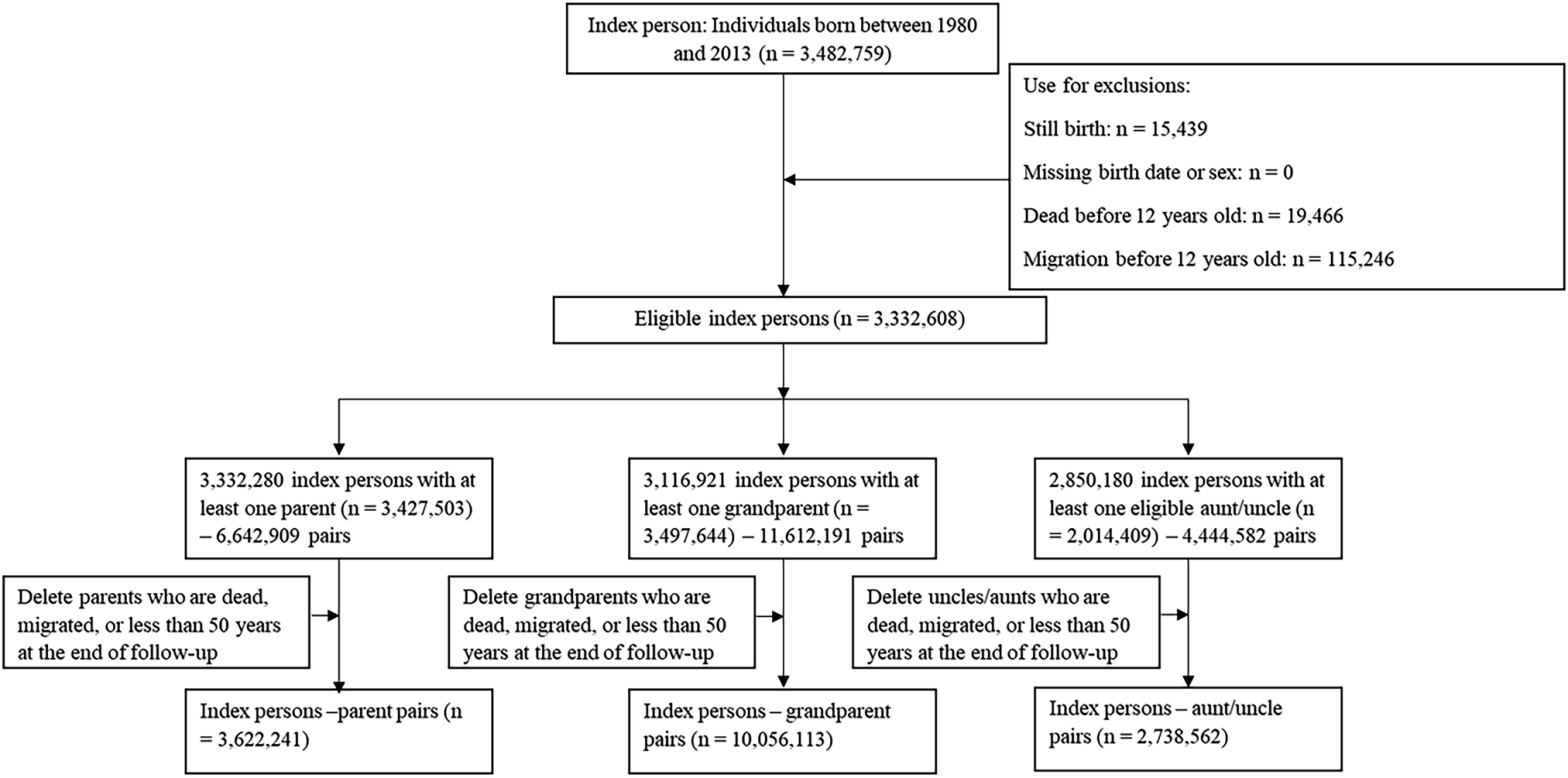
Flowchart of the inclusion of index persons and their biological relatives.

The cohort selection is shown in Fig. 1. We defined index individuals as individuals born between 1980–2013, identified from the MBR. We excluded individuals who were stillborn, missing information on birth date or sex, and individuals who died or migrated before age 12. We used the Multi-Generation Register to link each index person to their biological relatives, including parents, grandparents, and aunts or uncles. To ensure comparability between the parents and aunts/uncles, we only included aunts or uncles with at least one child and whose age was nearest to the age of the index individuals’ parents. We thus derived three cohorts of relatives: parents (who share 50% of their segregating DNA on average with the index individuals), grandparents (25% shared DNA), and aunts/uncles (25% shared DNA).

### Ethical approval and consent to participate

The study was approved by the Swedish Ethical Review Authority (2020-06540). This study was performed in accordance with Swedish laws concerning data privacy. Informed consent is not required for register-based studies in Sweden.

### Exposure and outcomes

We identified diagnoses of autism in index individuals from the NPR, using the specific ICD codes shown in Supplementary Table S1. We identified dementia in the relative cohorts based on the NPR or Cause of Death Register. In line with prior research, we defined dementia in three ways: any diagnosis of dementia, Alzheimer’s disease, and other dementias (aside from Alzheimer’s disease) [19]. The onset of dementia was approximated as either three years before the initial recorded diagnosis in the NPR or five-years before death (as identified from the Cause of Death Register), whichever came first. This approach to defining dementia was based on prior research [19, 20]. Codes used to identify dementia are shown in Supplementary Table S1. Covariates included sex of the index (which was the most recently recorded legal sex in the TPR) individuals and birth year of the index individuals and their relatives. Birth year was categorized into 1980–1989, 1990–2000, and after 2000 for index individuals. For relatives, five-year intervals were used, starting with the earliest possible birth year. Intellectual disability in the index individuals was identified from the NPR, using the ICD codes shown in Supplementary Table S1.

### Statistical analysis

For the main analyses, we analyzed the association between autism in index individuals and dementia in the three relative cohorts: parents, grandparents, and aunts/uncles. All relatives were followed up from age 50 to onset of dementia, migration, death, or the end of follow-up (December 31st 2020), whichever came first. We used a lifetime definition of autism (i.e. participants were considered autistic if they were diagnosed with autism at any point in their lives). We used Cox proportional hazards models with age of the relative as the underlying timescale. Hazard ratios (HRs) and 95% confidence intervals (CIs) were calculated, with standard errors clustered on maternal ID to account for related individuals in the analyses. We adjusted for sex of the index individuals, and birth year categories for index individuals and their relatives. We initially estimated the association between autism in index individuals and any diagnosis of dementia in their relatives, then conducted separate analyses of Alzheimer’s disease and other dementias.

To assess whether the observed associations differ with age of dementia onset, we subsequently performed secondary analyses for early onset dementia (onset prior to age 65) and later-onset dementia (onset after age 65). To assess sex differences in these associations, we conducted sex stratified analyses. We first stratified on the sex of the relatives and then the sex of the index individuals, in two separate models. Finally, we calculated separate HRs for relatives of autistic individuals with a diagnosis of intellectual disability and those without a diagnosis of intellectual disability.

We conducted several sensitivity analyses to test the robustness of our results. First, we repeated the analyses with the dementia onset defined as the date of the first dementia diagnosis, rather than three-years prior to diagnosis. Second, we included prescriptions of medication to define dementia (ATC codes are shown in Supplementary Table S1). The onset of dementia in these cases was estimated based on the date of the first relevant prescription documented in the PDR, consistent with prior studies [20]. Third, given the increased prevalence of autism diagnoses [21], we accounted for potential time trends in autism diagnoses by restricting the analysis to index individuals born between 1980–2005 in order to capture more people diagnosed prior to the sharp rise in autism diagnoses.

To further explore whether the familial association between autism and dementia was driven by genetic factors, we conducted two additional sensitivity analyses to examine the associations between number of individuals with an autism diagnosis in children and grandchildren and risk of dementia. The numbers of autistic individuals in children and grandchildren were divided into three groups (zero, single occurrence, and multiple occurrence [two or more]).

We used SAS version 9.4 for data management, and the survival package in R (version 4.2.3) for the statistical analyses.

## Results

Overall, we identified 3,332,608 index individuals, of whom 88,343 (2.65%, 57,619 male) had received a diagnosis of autism (Table 1). The parent cohort included 3,622,241 unique pairs of index individuals and parents. We also identified 10,056,113 index individual-grandparent pairs and 2,738,562 unique index individual-aunt/uncle pairs. Due to the younger age of the parent and aunt/uncle cohorts, the average age of dementia onset was lower than for grandparents.

**Table 1:** Descriptive characteristics of the three relative cohorts.

Cohorts	Variables	Overall	Men	Women
Index persons	No.	3,332,608	1,712,340 (51.38%)	1,620,268 (48.62%)
	Autism, n (%)	88,343 (2.65%)	57,619 (3.36%)	30,724 (1.90%)
	Intellectual disability, n (%)	37,321 (1.12%)	22,330 (1.30%)	14,991 (0.93%)
	Diagnosis age of autism, median (IQR)	15 (10, 20)	13 (9, 19)	16 (12, 21)
	Diagnosis age of ID, median (IQR)	12 (7, 17)	11 (7, 16)	13 (7, 18)
Parents	No. of relative pairs	3,622,241	1,886,705 (52.09%)	1,735,536 (47.91%)
	No. of relatives	1,802,180	937,591 (52.03%)	864,589 (47.97%)
	Age by the end of study, median (IQR)	61 (56, 67)	62 (56, 69)	60 (55, 66)
	Any dementia, n (%)	11,879 (0.66%)	7932 (0.85%)	3947 (0.46%)
	Alzheimer’s disease n (%)	9326 (0.52%)	6020 (0.64%)	3306 (0.38%)
	Other dementia, n (%)	4588 (0.25%)	3435 (0.37%)	1153 (0.13%)
	Any dementia, n (%)^b^	14,244 (0.79%)	9440 (1.01%)	4804 (0.56%)
	Alzheimer’s disease n (%)^b^	12,573 (0.70%)	8208 (0.88%)	4365 (0.50%)
	Onset age of any dementia, median (IQR)	66 (60, 71)	67 (61, 72)	63 (57, 68)
	Onset age of AD, median (IQR)	66 (60, 71)	67 (61, 73)	63 (58, 68)
	Onset age of other dementia, median (IQR)	66 (61, 71)	67 (62, 72)	63 (57, 67)
	Onset age of any dementia, median (IQR)^a^	69 (62, 74)	70 (64, 75)	66 (60, 71)
	Onset age of AD, median (IQR)^a^	69 (63, 74)	71 (64, 76)	66 (60, 71)
	Onset age of other dementia, median (IQR)^a^	69 (63, 74)	70 (65, 75)	65 (60, 70)
Grandparents	No. of unique pairs	10,056,113	4,985,137 (49.57%)	5,070,976 (50.43%)
	No. of relatives	3,032,259	1,499,774 (49.46%)	1,532,485 (50.54%)
	Age by the end of study, median (IQR)	84 (74, 96)	85 (75, 98)	82 (72, 95)
	Any dementia, n (%)	296,244 (9.77%)	128,202 (8.55%)	168,042 (10.97%)
	Alzheimer’s disease, n (%)	256,489 (8.46%)	107,422 (7.16%)	149,067 (9.73%)
	Other dementia, n (%)	86,370 (2.85%)	43,735 (2.92%)	42,635 (2.78%)
	Any dementia, n (%)^b^	320,272 (10.56%)	138,046 (9.20%)	182,226 (11.89%)
	Alzheimer’s disease, n (%)^b^	288,076 (9.50%)	121,339 (8.09%)	166,737 (10.88%)
	Onset age of any dementia, median (IQR)	79 (74, 84)	78 (73, 83)	80 (74, 85)
	Onset age of AD, median (IQR)	79 (74, 84)	78 (73, 83)	80 (75, 85)
	Onset age of other dementia, median (IQR)	78 (73, 83)	77 (72, 82)	79 (74, 84)
	Onset age of any dementia, median (IQR)^a^	83 (77, 88)	82 (76, 86)	84 (78, 88)
	Onset age of AD, median (IQR)^a^	83 (77, 88)	82 (77, 87)	84 (78, 89)
	Onset age of other dementia, median (IQR)^a^	82 (76, 87)	80 (75, 85)	83 (77, 88)
Aunts/uncles	No. of unique pairs	2738,562	1,345,089 (49.12%)	1,393,473 (50.88%)
	No. of relatives	1,208,371	592,419 (49.02%)	615,952 (50.97%)
	Age by the end of study, median (IQR)	62 (56, 69)	62 (56, 69)	62 (56, 69)
	Any dementia, n (%)	10,486 (0.87%)	5247 (0.89%)	5239 (0.85%)
	AD, n (%)	8443 (0.70%)	4001 (0.68%)	4442 (0.72%)
	Other dementia, n (%)	3669 (0.30%)	2194 (0.37%)	1475 (0.24%)
	Any dementia, n (%)^b^	12,772 (1.06%)	6348 (1.07%)	6424 (1.04%)
	AD, n (%)^b^	11,455 (0.95%)	5566 (0.94%)	5889 (0.96%)
	Onset age of any dementia, median (IQR)	67 (62, 72)	67 (62, 72)	68 (61, 72)
	Onset age of AD, median (IQR)	68 (62, 72)	68 (62, 73)	68 (62, 72)
	Onset age of other dementia, median (IQR)	67 (62, 72)	67 (62, 72)	67 (61, 72)
	Onset age of any dementia, median (IQR)^a^	71 (64, 76)	70 (65, 75)	71 (64, 76)
	Onset age of AD, median (IQR)^a^	71 (65, 76)	71 (65, 76)	71 (65, 76)
	Onset age of other dementia, median (IQR)^a^	70 (64, 75)	70 (65, 75)	70 (64, 76)

The numbers in this table are based on the unique relatives.

*AD* Alzheimer’s disease, *ID* intelligence disability, *IQR* interquartile range.

^a^Define onset of dementia based on the diagnosis date.

^b^Define by both dementia diagnosis and medication.

### Autism and dementia across generations

**Fig. 2:**
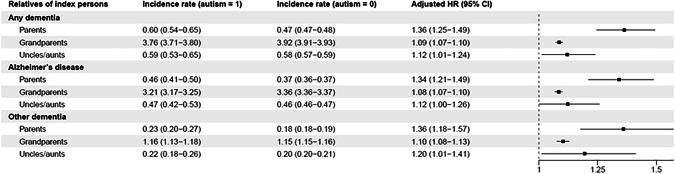
Associations between autism and dementia across generations. Incidence rates are shown for index person–relative pairs (not individuals) and are per 1000 person years; adjusted hazard ratios were derived from Cox proportional models adjusted for sex of index person index birth year categories, and relative birth year categories. The forest plot employs a logarithmic scale for the adjusted *HR* and its associated 95% confidence interval. *CI* confidence interval, *HR* hazard ratio.

The incidence rate of dementia was higher in the parents of autistic individuals (0.60 per 1000 person years) than in parents of nonautistic individuals (0.47 per 1000 person years); see Fig. 2 and Supplementary Table S2. All hazards ratios (HRs) presented in this section are adjusted for sex of the index individuals and birth year of index individuals and their relatives. Parents of autistic individuals were at an increased risk of any dementia (HR = 1.36, 95% CI = 1.25–1.49). This association attenuated in grandparents (HR = 1.09, 1.07–1.10) and aunts/uncles (HR = 1.12, 1.01–1.24), albeit the confidence intervals overlapped for aunts/uncles and parents. Similar patterns of results were observed for Alzheimer’s disease; parents of autistic individuals were at an increased risk for Alzheimer’s disease (HR = 1.34, 1.21–1.49), while the risk increase was lower in grandparents (HR = 1.08, 1.07–1.10) and aunts/uncles (HR = 1.12, 1.00–1.26). Similarly, parents were at an increased risk for other types of dementia (HR = 1.36, 1.18–1.57). This was attenuated in aunts/uncles and grandparents (Fig. 2).

The results for early- and later-onset dementia are shown in Supplementary Table S3. Although there was overlap in the confidence intervals, the HRs were stronger for early-onset dementia compared to later-onset dementia.

### Sex-stratified analyses

**Fig. 3:**
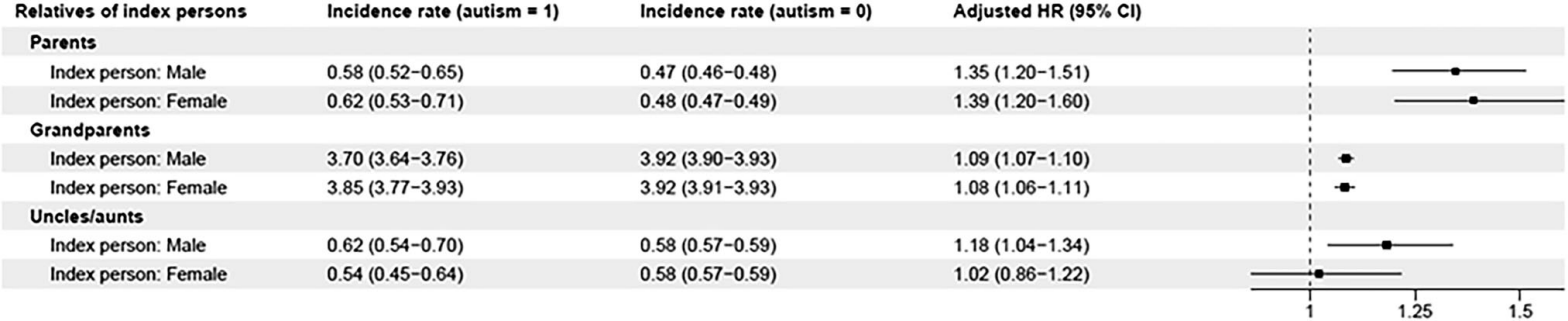
Associations between autism and dementia across generations, stratified by sex of index persons. Incidence rates are shown for index person–relative pairs (not individuals); adjusted hazard ratios were derived from Cox proportional models adjusted for index birth year categories and relative birth year categories. The forest plot employs a logarithmic scale for the adjusted *HR* and its associated 95% confidence interval. *CI* confidence interval, *HR* hazard ratio.

For any diagnosis of dementia, the associations were generally similar between women and men (Fig. 3 and Supplementary Table S4). Parents of autistic females (HR = 1.39, 1.20–1.60) and males (HR = 1.35, 1.20–1.51) were at a similarly increased relative risk of dementia. Associations were attenuated in grandparents and were similar in both sexes (Fig. 3). In aunts/uncles, the relatives of autistic males (HR = 1.18, 1.04–1.34) were at a greater relative risk of dementia than the relatives of autistic females (HR = 1.02, 0.86–1.22), although confidence intervals overlapped. Similar results were observed for Alzheimer’s disease and other types of dementia (Supplementary Table S4)

When stratifying on the sex of the relatives, there was some evidence of a stronger association between autism and dementia in mothers (HR = 1.51, 1.29–1.77) than in fathers (HR = 1.30, 1.16–1.45); see Supplementary Table S2. However, a similar pattern was not observed in the sex stratified analyses of grandparents or aunts/uncles. Grandmothers (HR = 1.08, 1.06–1.10) and grandfathers (HR = 1.09, 1.07–1.11) of autistic individuals were at a similarly increased risk of dementia. The same was true for aunts (HR = 1.10, 0.96–1.28) and uncles (HR = 1.14, 0.98–1.31) of autistic individuals. The same pattern of results was observed for Alzheimer’s disease and other types of dementia (Supplementary Table S2).

### Intellectual disability

**Fig. 4:**
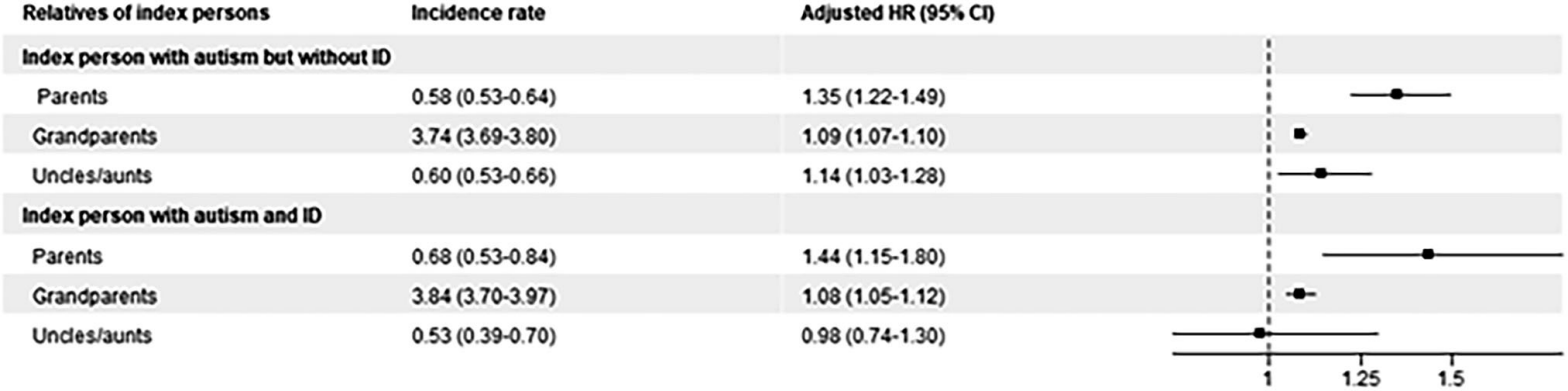
Associations between autism and any dementia across generations, stratified by intellectual disability of the index persons. Index-parents pairs: 76,440 index individuals with autism without ID, 12,299 with both autism and ID. Index-grandparents pairs: 225,750 index individuals with only autism, 34,700 with both autism and ID. Index-uncle/aunts pairs: 56,040 index individuals with only autism, 7026 with both autism and ID. Incidence rates are shown for index person–relative pairs (not individuals); adjusted hazard ratios were derived from Cox proportional models adjusted for index sex, index birth year categories, and relative birth year categories, and the reference group is individuals without autism. The forest plot employs a logarithmic scale for the adjusted *HR* and its associated 95% confidence interval. *CI* confidence interval, *HR* hazard ratio, *ID* intelligence disability.

There was a higher incidence rate of dementia in parents of autistic individuals with intellectual disability (0.68 per 1000 person years) than in parents of those without an intellectual disability (0.58 per 1000 person years). The parents of autistic individuals with (HR = 1.44, 1.15–1.80) and without intellectual disability (HR = 1.35, 1.22–1.49) were both at a higher relative risk for dementia (Fig. 4). The results were similar for Alzheimer’s disease, other dementias, and sex-stratified analyses (Supplementary Tables S5 and S6).

### Sensitivity analyses

Defining dementia onset based on the date of first dementia diagnosis, defining dementia based on prescriptions of medication, and restricting the analyses to relatives of individuals born from 1980–2005 did not alter the results (Supplementary Tables S7–S9). When examining the number of autistic individuals in children and grandchildren, (Supplementary Table S10), there was a stronger association between autism and dementia in parents for individuals from multiple occurrence families (HR = 1.39, 1.09–1.77) compared to individuals from single occurrence families (HR = 1.13, 1.07–1.18), albeit the sample size was somewhat small for multiple occurrence families and confidence intervals overlapped. Associations attenuated in grandparents, similar to the main analyses.

## Discussion

To our knowledge, this is the first study to investigate the familial association between autism and dementia. Overall, we found that relatives of autistic individuals were at an increased risk of dementia. This was particularly the case for parents of autistic individuals compared to grandparents and aunts or uncles. In other words, the association between autism in index individuals and dementia in relatives was stronger for more closely related individuals. The same pattern of results was observed for Alzheimer’s disease and other types of dementia. The results therefore indicate that autism and dementia may be underpinned by an overlapping set of familial factors.

It is not possible to determine whether the increased incidence rate and risk of dementia in parents of autistic individuals is driven by genetic factors or by shared environment (i.e. environmental factors shared between individuals residing in the same home). However, it is worth highlighting that the familial association between autism and dementia was stronger in families where two or more individuals had received a diagnosis of autism. Autism in such a context is more likely to be linked with an inherited genetic etiology, and consequently this result could be indicative of shared genetic influences between autism and dementia. In addition, twin studies of autism consistently suggest that shared environment plays little to no role in autism [22]. Thus, it is entirely possible that the observed associations here were driven by genetic factors. There are several ways in which autism and dementia may be genetically linked. There could be an indirect genetic link between autism and dementia, whereby autism itself mediates the association between the genetic influences on autism and dementia. This was not supported by a Mendelian randomization study, however, albeit the autism GWAS on which this study was based had a small sample size [12]. Our results may also reflect pleiotropic genetic links between autism and dementia. To date, however, there remains limited evidence to support this hypothesis. The most recent GWAS of autism and Alzheimer’s disease did not report significant genetic correlations between these conditions [10, 11]. This may reflect the relatively low statistical power of the current autism GWAS. It may also be the case that rare variants drive the association between autism and dementia. Given that our cohort of parents of autistic individuals captured more early onset dementias, this is a potentially important consideration in future, given that earlier onset dementias have been linked with rare variants [23].

A caveat of the above discussion is that we cannot fully distinguish between genetic and shared environmental influences on the association between autism and dementia in this study. While children share more segregating DNA with their parents than with a grandparent or aunt/uncle, they also share more of their home environment. While the shared environmental contribution to autism appears negligible [22], it is possible that such factors could still account for the association between autism and dementia. The Lancet commission on dementia highlighted a number of pertinent risk factors for dementia, many of which may be shared by autistic individuals and relatives with whom they share the same home [24]. For example, autistic individuals and their relatives are known to be at an increased risk of depression, which was highlighted as a risk factor for dementia [25]. Researchers have also highlighted stress as a potential, under-researched risk factor for dementia [26]. Autistic individuals may face more stress as they navigate a world that is not matched to their needs, and their parents may experience stress while advocating for them. Future research should thus clarify whether experiences shared by autistic individuals and their families contribute to the risk of dementia in the families of autistic individuals.

Our finding that autism and dementia may be genetically linked adds to the growing overall picture of shared genetic causes between autism and other conditions. Autism regularly co-occurs with psychiatric disorders and other neurodevelopmental conditions [27]. Twin and family studies robustly show genetic correlations between autism, attention-deficit/hyperactivity disorder, anxiety disorders, and depression [25]. Molecular genetic studies are starting to show significant genetic correlations between autism and other conditions, such as ADHD and depression, in terms of common variants [10], although there remains scant evidence concerning the etiological links between autism and neurological conditions. Our results therefore indicate that the genetic links shown between autism, other neurodevelopmental conditions, and psychiatric disorders may extend to dementia. This highlights that genetic causes of autism may cut across not only psychiatric and neurodevelopmental conditions, but also neurological disorders.

Given that we did not investigate the risk of dementia in autistic individuals directly, we caution against drawing strong conclusions about the individual risk of dementia in autistic individuals from our results. Cohorts of older autistic individuals are subject to various biases, such as survival bias (i.e. they are likely to include healthier autistic individuals, given the increased rate of premature mortality in autistic individuals), and selection biases (i.e. older individuals being less likely to have received an autism diagnosis). Such factors may lead to an under-estimation of the association between autism and dementia. In addition, autism is mostly diagnosed in outpatient settings in Sweden and we only have coverage of outpatient care since 2001, meaning it would be difficult to reliably identify autism diagnoses for older members of our cohort. While we do not draw strong conclusions here concerning the risk of dementia in autistic individuals, our results should be taken together with emerging studies showing increased rates of cognitive decline and dementia in autistic individuals [8, 9]. More studies are needed to clarify the risk of dementia in autistic individuals.

Our stratified results (on sex and intellectual disability) were based on smaller sample sizes and conclusions should be drawn with caution. However, one result that was consistent across analyses was a higher increased risk of dementia in mothers of autistic individuals compared to fathers. This is similar to studies of autism and other conditions, such as asthma, where a stronger maternal effect has been found [28]. Such an effect could be caused by shared environmental factors, as discussed above. It is also worth highlighting that maternity is better covered in our registry data than paternity.

When stratifying the analyses on intellectual disability in autistic individuals, we found only minor differences in the associations with dementia in relatives. There was a higher incidence rate of dementia in relatives of autistic individuals with intellectual disability compared to those without, yet the risk was increased in the relatives of all autistic individuals. It is difficult to determine whether this pattern of results is to be expected or not, given that there is limited evidence concerning dementia in individuals with intellectual disability [29]. Prior studies have suggested that autistic individuals with intellectual disability are at a higher risk for neurological disorders more broadly, which may mean that our findings could be seen as unexpected [30]. More research is evidently needed on dementia in individuals with intellectual disability in order to better understand these results.

### Strengths and limitations

Strengths of our study include the use of a large, population-based cohort. We were able to study the inter-generational associations between autism and dementia across multiple generations. Our large sample also enabled us to conduct a series of stratified analyses and sensitivity analyses to assess the robustness of our results. There are limitations to consider, however. As mentioned above, we did not estimate the risk of dementia in autistic individuals due to lack of extensive follow-up of older autistic individuals in our sample, an issue which has presented challenges in the field more broadly. Future longitudinal studies of representative cohorts of autistic individuals are needed to assess whether autistic individuals are at an increased risk of dementia. It is also important to highlight that our study is based on data recorded about Swedish healthcare. While it is an advantage that Sweden has a universal healthcare system, the results do not necessarily generalize to countries with different healthcare systems. It is therefore important for more studies to be conducted to replicate our results, particularly given the dearth of available research on autism and dementia. A further important limitation of our study is that the parent cohort had less follow-up. Due to the relatively young age of this cohort compared to the grandparents, the prevalence of dementia was very low in these individuals. Furthermore, there were fewer individuals with late onset dementia due to the age of the cohort, meaning that later onset dementias were not as well captured in this cohort. Finally, we did not account for autism in relatives in these analyses. Relatives of autistic individuals are substantially more likely to be autistic than relatives of nonautistic individuals. Thus, if a within-individual association between autism and dementia were to exist (which we did not assess here), then this may inflate the risk of dementia in relatives of autistic individuals. Due to time trends in autism diagnoses and coverage of outpatient care in our data, it is unlikely that autism is as well covered in these relatives, with median ages of 61 to 84 at the end of follow-up, making such an adjustment challenging. Future studies should thus clarify whether the familial association between autism and dementia is driven by the within-individual between autism and dementia in relatives.

## Conclusion

To conclude, our study provides some of the first evidence to suggest that autism and dementia co-aggregate within families. This lends support to the hypothesis that these conditions are underpinned by shared genetic factors, thus indicating that the genetics of neurodevelopmental and neurodegenerative conditions may be linked. These results require replication, and it is important to also clarify the risk of dementia in autistic individuals themselves, as well as investigating the potential shared genetic basis of these conditions.

## Supplementary information

Supplementary material

## Data Availability

Due to Swedish legislation concerning data privacy, we are unable to share data from this study with others. Researchers interested in accessing the data are able to apply for access from the Swedish National Board of Health and Welfare and Statistics Sweden.

## Code availability

We are willing to share analytic code on request.
