# Supplementary material for: Association between autism and dementia across generations: evidence from a family study of the Swedish population
Source: Mol Psychiatry. 2025 May 14;30(10):4605–12. doi: 10.1038/s41380-025-03045-6 (PMC12436158; doi:10.1038/s41380-025-03045-6)
Supplement: Supplementary file 1 — Supplementary material [file 41380_2025_3045_MOESM1_ESM.docx]

**Supplementary Materials**

**Article:** Association between autism and dementia across generations: evidence from a family study of the Swedish population

**Authors:** Zheng Chang, Honghui Yao, Shihua Sun, Le Zhang, Shengxin Liu, Isabell Brikell, Brian M. D’Onofrio, Henrik Larsson, Paul Lichtenstein, Ralf Kuja-Halkola, Sara Hägg, Francesca Happé, & Mark J. Taylor

**Table of Contents**

Page 2. Supplementary Table S1 ICD and ATC codes used in the study

Page 4. Supplementary Table S2 Associations between autism and any dementia/Alzheimer's disease/other dementia across generations

Page 6. Supplementary Table S3 Associations between autism and early/late onset any dementia across generations

Page 7. Supplementary Table S4 Associations between autism and any dementia/Alzheimer's disease/other dementia across generations stratified by index sex

Page 9. Supplementary Table S5 The incidence of any dementia/Alzheimer's disease/other dementia across generations stratified by the intellectual disability of index persons

Page 10. Supplementary Table S6 Associations between autism and any dementia/Alzheimer's disease/other dementia across generations stratified by intellectual disability in index persons

Page 12. Supplementary Table S7 Sensitivity analysis: associations between autism and any dementia/Alzheimer's disease/other dementia across generations: refining diagnosis and death timing in dementia

Page 14. Supplementary Table S8 Sensitivity analysis: associations between autism and any dementia/Alzheimer's disease/other dementia across generations after adding Alzheimer's disease medication to define outcomes

Page 15. Supplementary Table S9 Sensitivity analysis: associations between autism and any dementia/Alzheimer's disease/other dementia across generations among index individuals born between 1980 and 2005

Page 17. Supplementary Table S10 Distribution of dementia cases across varying numbers of autistic family members

Page 17. Supplementary Table S11 Association between autism and dementia in relatives stratified on number of autistic individuals in the family

**Supplementary Table S1** ICD and ATC codes used in the study

|  | **ICD-8** | **ICD-9** | **ICD-10** | **ATC** |
| --- | --- | --- | --- | --- |
| Years | 1969-1986 | 1987-1996 | 1997-2020 | 2005-2020 |
| Autism |  | 299A (infantile autism) | F84.0 (childhood autism), F84.1 (atypical autism), F84.5 (Asperger syndrome), F84.8 (other pervasive developmental disorders), F84.9 (pervasive developmental disorder, unspecified) |  |
| Intellectual disability | 311 (mild intellectual disability); 312 (moderate intellectual disability); 313 (severe intellectual disability); 314 (profound intellectual disability); 315 (unspecified intellectual disability) | 317 (mild intellectual disability); 318 (moderate, severe, or profound intellectual disability); 319 (unspecified intellectual disability) | F70 (mild intellectual disability); F71 (moderate intellectual disability); F72 (severe intellectual disability); F73 (profound intellectual disability); F78 (other intellectual disability); F79 (unspecified intellectual disability) |  |
| Alzheimer’s disease | 290 (senile dementia) | 290A (senile dementia), 290B (presenile dementia), 290X (unspecified dementia associated with age), 331A (presenile and senile dementia of the Alzheimer type) | F00 (dementia in Alzheimer disease), F03 unspecified dementia), G30 (Alzheimer disease) | N06DA02 (donepezil hydrochloride), N06DA03 (rivastigmine), N06DA04 (galantamine), N06DX01 (memantine) |
| Other dementia | 293.0 (psychosis associated with cerebral arteriosclerosis); 293.1 (psychosis associated with another cerebrovascular disease) | 290E (multi-infarct dementia), 290W (other specific dementia associated with age), 294B (dementia in somatic disease classified elsewhere), 331B (Pick’s disease), 331C (unspecified senile degeneration of the brain, unspecified type), 331X (unspecified cerebral degeneration) | F01 (vascular dementia), F02 (dementia in other diseases classified elsewhere), F05.1 (delirium superimposed on dementia), G31.1 (senile degeneration of the brain, not elsewhere classified), G31.8 (other specified degenerative diseases of the nervous system) |  |

**Supplementary Table S2** Associations between autism and any dementia/Alzheimer's disease/other dementia across generations

| **Relative cohorts** | **Autistic index person** | | **Nonautistic index person** | | **Crude HR**  **(95% CI)** | **Adjusted HR**  **(95% CI)*** |
| --- | --- | --- | --- | --- | --- | --- |
|  | **No. of events** | **Incidence rate** | **No. of events** | **Incidence rate** |  |  |
| **Any dementia** | | | | | | |
| Parents | 482 | 0.60 (0.54-0.65) | 17,666 | 0.47 (0.47-0.48) | 1.35 (1.23-1.47) | 1.36 (1.25-1.49) |
| Mother | 157 | 0.45 (0.38-0.53) | 5,707 | 0.34 (0.34-0.35) | 1.43 (1.22-1.67) | 1.51 (1.29-1.77) |
| Father | 325 | 0.70 (0.63-0.78) | 11,959 | 0.58 (0.57-0.59) | 1.29 (1.16-1.45) | 1.30 (1.16-1.45) |
| Grandparents | 23,822 | 3.76 (3.71-3.80) | 974,872 | 3.92 (3.91-3.93) | 1.06 (1.04-1.07) | 1.09 (1.07-1.10) |
| Grandmother | 12,556 | 3.87 (3.80-3.94) | 532,897 | 4.16 (4.15-4.18) | 1.05 (1.03-1.07) | 1.08 (1.06-1.10) |
| Grandfather | 11,266 | 3.64 (3.57-3.70) | 441,975 | 3.66 (3.65-3.67) | 1.07 (1.05-1.09) | 1.09 (1.07-1.11) |
| Uncles/aunts | 382 | 0.59 (0.53-0.65) | 17,481 | 0.58 (0.57-0.59) | 1.11 (1.00-1.23) | 1.12 (1.01-1.24) |
| Aunt | 189 | 0.56 (0.49-0.65) | 8,709 | 0.57 (0.55-0.58) | 1.09 (0.95-1.26) | 1.10 (0.96-1.28) |
| Uncle | 193 | 0.61 (0.53-0.71) | 8,772 | 0.60 (0.58-0.61) | 1.12 (0.97-1.29) | 1.14 (0.98-1.31) |
| **Alzheimer’s disease** | | | | | | |
| Parents | 369 | 0.46 (0.41-0.50) | 13,750 | 0.37 (0.36-0.37) | 1.32 (1.19-1.47) | 1.34 (1.21-1.49) |
| Mother | 133 | 0.38 (0.32-0.45) | 4,742 | 0.29 (0.28-0.29) | 1.46 (1.23-1.74) | 1.54 (1.30-1.83) |
| Father | 236 | 0.51 (0.45-0.58) | 9,008 | 0.43 (0.43-0.44) | 1.24 (1.09-1.42) | 1.25 (1.10-1.42) |
| Grandparents | 20,423 | 3.21 (3.17-3.25) | 840,274 | 3.36 (3.36-3.37) | 1.05 (1.04-1.07) | 1.08 (1.07-1.10) |
| Grandmother | 11,061 | 3.40 (3.34-3.46) | 471,035 | 3.67 (3.66-3.68) | 1.05 (1.03-1.07) | 1.08 (1.06-1.10) |
| Grandfather | 9,362 | 3.01 (2.95-3.07) | 369,239 | 3.04 (3.03-3.05) | 1.07 (1.05-1.09) | 1.09 (1.06-1.11) |
| Uncles/aunts | 306 | 0.47 (0.42-0.53) | 13,992 | 0.46 (0.46-0.47) | 1.11 (0.99-1.24) | 1.12 (1.00-1.26) |
| Aunt | 161 | 0.48 (0.41-0.56) | 7,365 | 0.48 (0.47-0.49) | 1.10 (0.94-1.29) | 1.12 (0.96-1.31) |
| Uncle | 145 | 0.46 (0.39-0.54) | 6,627 | 0.45 (0.44-0.46) | 1.12 (0.95-1.32) | 1.13 (0.96-1.33) |
| **Other dementia** | | | | | | |
| Parents | 189 | 0.23 (0.20-0.27) | 6,870 | 0.18 (0.18-0.19) | 1.36 (1.18-1.57) | 1.36 (1.18-1.57) |
| Mother | 49 | 0.14 (0.10-0.19) | 1,658 | 0.10 (0.10-0.10) | 1.53 (1.15-2.03) | 1.64 (1.23-2.17) |
| Father | 140 | 0.30 (0.25-0.36) | 5,212 | 0.25 (0.24-0.26) | 1.28 (1.09-1.52) | 1.28 (1.08-1.51) |
| Grandparents | 7,452 | 1.16 (1.13-1.18) | 292,232 | 1.15 (1.15-1.16) | 1.09 (1.06-1.11) | 1.10 (1.08-1.13) |
| Grandmother | 3,430 | 1.04 (1.00-1.07) | 138,015 | 1.06 (1.05-1.06) | 1.10 (1.06-1.13) | 1.12 (1.09-1.16) |
| Grandfather | 4,022 | 1.28 (1.24-1.32) | 154,217 | 1.26 (1.25-1.26) | 1.08 (1.05-1.11) | 1.08 (1.05-1.12) |
| Uncles/aunts | 141 | 0.22 (0.18-0.26) | 6,103 | 0.20 (0.20-0.21) | 1.17 (0.99-1.39) | 1.20 (1.01-1.41) |
| Aunt | 51 | 0.15 (0.11-0.20) | 2,469 | 0.16 (0.15-0.17) | 1.04 (0.79-1.37) | 1.05 (0.79-1.38) |
| Uncle | 90 | 0.29 (0.23-0.35) | 3,634 | 0.25 (0.24-0.25) | 1.27 (1.03-1.56) | 1.30 (1.05-1.60) |

CI, confidence interval; HR, hazard ratio

* Adjusted for index sex, index birth year categories, and relative birth year categories

**Supplementary Table S3** Associations between autism and early/late onset any dementia across generations

| **Relative cohorts** | **Autistic index person** | | **Nonautistic index person** | | **Crude HR**  **(95% CI)** | **Adjusted HR**  **(95% CI)*** |
| --- | --- | --- | --- | --- | --- | --- |
|  | **No. of events** | **Incidence rate** | **No. of events** | **Incidence rate** |  |  |
| **Early onset any dementia** | | | | | | |
| Parents | 270 | 0.37 (0.33-0.42) | 9,526 | 0.29 (0.28-0.29) | 1.35 (1.20-1.53) | 1.45 (1.28-1.63) |
| Mother | 116 | 0.36 (0.30-0.43) | 3,891 | 0.26 (0.25-0.27) | 1.47 (1.22-1.77) | 1.59 (1.32-1.92) |
| Father | 154 | 0.38 (0.32-0.44) | 5,635 | 0.32 (0.31-0.32) | 1.27 (1.08-1.49) | 1.35 (1.15-1.58) |
| Grandparents | 2,059 | 0.56 (0.54-0.59) | 66,129 | 0.48 (0.47-0.48) | 1.18 (1.13-1.24) | 1.16 (1.11-1.21) |
| Grandmother | 1,040 | 0.56 (0.52-0.59) | 32,959 | 0.47 (0.46-0.47) | 1.20 (1.12-1.27) | 1.18 (1.11-1.26) |
| Grandfather | 1,019 | 0.57 (0.53-0.60) | 33,17 | 0.49 (0.48-0.49) | 1.17 (1.10-1.25) | 1.14 (1.07-1.21) |
| Uncles/aunts | 193 | 0.34 (0.29-0.39) | 7,847 | 0.31 (0.30-0.31) | 1.16 (1.01-1.34) | 1.22 (1.06-1.41) |
| Aunt | 101 | 0.34 (0.28-0.42) | 3,806 | 0.29 (0.28-0.30) | 1.23 (1.01-1.50) | 1.30 (1.07-1.59) |
| Uncle | 92 | 0.34 (0.27-0.41) | 4,041 | 0.32 (0.31-0.33) | 1.09 (0.89-1.34)* | 1.14 (0.93-1.40) |
| **Late onset any dementia** | | | | | | |
| Parents | 212 | 2.60 (2.26-2.97) | 8,140 | 1.87 (1.83-1.91) | 1.34 (1.17-1.53) | 1.27 (1.11-1.46) |
| Mother | 41 | 1.59 (1.14-2.16) | 1,816 | 1.20 (1.15-1.26) | 1.32 (0.97-1.80) | 1.33 (0.98-1.81) |
| Father | 171 | 3.06 (2.62-3.55) | 6,324 | 2.23 (2.17-2.29) | 1.32 (1.13-1.53) | 1.26 (1.08-1.46) |
| Grandparents | 21,763 | 8.13 (8.03-8.24) | 908,743 | 8.26 (8.24-8.27) | 1.05 (1.03-1.06) | 1.08 (1.06-1.09) |
| Grandmother | 11,516 | 8.36 (8.21-8.51) | 499,938 | 8.69 (8.66-8.71) | 1.04 (1.02-1.06) | 1.08 (1.06-1.10) |
| Grandfather | 10,247 | 7.90 (7.74-8.05) | 408,805 | 7.79 (7.76-7.81) | 1.06 (1.04-1.08) | 1.08 (1.06-1.10) |
| Uncles/aunts | 189 | 2.27 (1.96-2.62) | 9,634 | 2.15 (2.10-2.19) | 1.06 (0.91-1.22) | 1.03 (0.89-1.19) |
| Aunt | 88 | 2.05 (1.64-2.52) | 4,903 | 2.11 (2.05-2.17) | 0.97 (0.78-1.19) | 0.94 (0.76-1.16) |
| Uncle | 101 | 2.51 (2.04-3.05) | 4,731 | 2.18 (2.12-2.24) | 1.15 (0.94-1.40) | 1.13 (0.93-1.39) |

CI, confidence interval; HR, hazard ratio

* Adjusted for index sex, index birth year categories, and relative birth year categories

**Supplementary Table S4** Associations between autism and any dementia/Alzheimer's disease/other dementia across generations stratified by index sex

| **Relative cohorts** | **Autistic index person** | | **Nonautistic index person** | | **Crude HR**  **(95% CI)** | **Adjusted HR**  **(95% CI)*** |
| --- | --- | --- | --- | --- | --- | --- |
|  | **No. of events** | **Incidence rate** | **No. of events** | **Incidence rate** |  |  |
| **Any dementia** | | | | | | |
| Parents |  |  |  |  |  |  |
| Index person: Male | 291 | 0.58 (0.52-0.65) | 8,985 | 0.47 (0.46-0.48) | 1.33 (1.19-1.50) | 1.35 (1.20-1.51) |
| Index person: Female | 191 | 0.62 (0.53-0.71) | 8,681 | 0.48 (0.47-0.49) | 1.37 (1.19-1.58) | 1.39 (1.20-1.60) |
| Grandparents |  |  |  |  |  |  |
| Index person: Male | 15,030 | 3.70 (3.64-3.76) | 497,612 | 3.92 (3.90-3.93) | 1.06 (1.04-1.07) | 1.09 (1.07-1.10) |
| Index person: Female | 8,792 | 3.85 (3.77-3.93) | 477,260 | 3.92 (3.91-3.93) | 1.06 (1.04-1.09) | 1.08 (1.06-1.11) |
| Uncles/aunts |  |  |  |  |  |  |
| Index person: Male | 250 | 0.62 (0.54-0.70) | 8,955 | 0.58 (0.57-0.59) | 1.16 (1.03-1.32) | 1.18 (1.04-1.34) |
| Index person: Female | 132 | 0.54 (0.45-0.64) | 8,526 | 0.58 (0.57-0.59) | 1.01 (0.85-1.20) | 1.02 (0.86-1.22) |
| **Alzheimer’s disease** | | | | | | |
| Parents |  |  |  |  |  |  |
| Index person: Male | 216 | 0.43 (0.38-0.49) | 6,986 | 0.37 (0.36-0.37) | 1.27 (1.11-1.45) | 1.28 (1.12-1.47) |
| Index person: Female | 153 | 0.49 (0.42-0.58) | 6,764 | 0.37 (0.36-0.38) | 1.41 (1.20-1.66) | 1.43 (1.22-1.68) |
| Grandparents |  |  |  |  |  |  |
| Index person: Male | 12,872 | 3.16 (3.11-3.22) | 429,014 | 3.36 (3.35-3.37) | 1.05 (1.03-1.07) | 1.08 (1.06-1.10) |
| Index person: Female | 7,551 | 3.30 (3.22-3.37) | 411,260 | 3.37 (3.36-3.38) | 1.06 (1.04-1.09) | 1.08 (1.06-1.11) |
| Uncles/aunts |  |  |  |  |  |  |
| Index person: Male | 199 | 0.49 (0.43-0.56) | 7,144 | 0.46 (0.45-0.47) | 1.16 (1.01-1.34) | 1.18 (1.03-1.36) |
| Index person: Female | 107 | 0.44 (0.36-0.53) | 6,848 | 0.46 (0.45-0.48) | 1.02 (0.84-1.24) | 1.03 (0.85-1.25) |
| **Other dementia** | | | | | | |
| Parents |  |  |  |  |  |  |
| Index person: Male | 120 | 0.24 (0.20-0.29) | 3,493 | 0.18 (0.18-0.19) | 1.43 (1.19-1.71) | 1.41 (1.17-1.69) |
| Index person: Female | 69 | 0.22 (0.17-0.28) | 3,377 | 0.18 (0.18-0.19) | 1.27 (1.00-1.61) | 1.28 (1.01-1.62) |
| Grandparents |  |  |  |  |  |  |
| Index person: Male | 4,739 | 1.15 (1.12-1.18) | 149,504 | 1.16 (1.15-1.16) | 1.09 (1.06-1.12) | 1.11 (1.08-1.14) |
| Index person: Female | 2,713 | 1.17 (1.12-1.21) | 142,728 | 1.15 (1.15-1.16) | 1.08 (1.04-1.12) | 1.09 (1.05-1.13) |
| Uncles/aunts |  |  |  |  |  |  |
| Index person: Male | 95 | 0.23 (0.19-0.29) | 3,154 | 0.20 (0.20-0.21) | 1.26 (1.03-1.54) | 1.28 (1.05-1.57) |
| Index person: Female | 46 | 0.19 (0.14-0.25) | 2,949 | 0.20 (0.19-0.21) | 1.02 (0.76-1.36) | 1.05 (0.78-1.40) |

CI, confidence interval; HR, hazard ratio

* Adjusted for index birth year categories and relative birth year categories

**Supplementary Table S5** The incidence of any dementia/Alzheimer's disease/other dementia across generations stratified by the intellectual disability of index persons

| **Relative cohorts** | **Nonautistic index person** | | **Autistic index person without ID** | | **Autistic index person with ID** | |
| --- | --- | --- | --- | --- | --- | --- |
|  | **No. of events** | **Incidence rate** | **No. of events** | **Incidence rate** | **No. of events** | **Incidence rate** |
| **Any dementia** | | | | | | |
| Parents | 17,666 | 0.47 (0.47-0.48) | 404 | 0.58 (0.53-0.64) | 78 | 0.68 (0.53-0.84) |
| Mother | 5,707 | 0.34 (0.34-0.35) | 136 | 0.46 (0.38-0.54) | 21 | 0.43 (0.27-0.66) |
| Father | 11,959 | 0.58 (0.57-0.59) | 268 | 0.68 (0.60-0.76) | 57 | 0.86 (0.65-1.11) |
| Grandparents | 974,872 | 3.92 (3.91-3.93) | 20,573 | 3.74 (3.69-3.80) | 3,249 | 3.84 (3.70-3.97) |
| Grandmother | 532,897 | 4.16 (4.15-4.18) | 10,828 | 3.85 (3.78-3.93) | 1,728 | 3.99 (3.81-4.19) |
| Grandfather | 441,975 | 3.66 (3.65-3.67) | 9,745 | 3.63 (3.56-3.70) | 1,521 | 3.67 (3.49-3.86) |
| Uncles/aunts | 17,481 | 0.58 (0.57-0.59) | 333 | 0.60 (0.53-0.66) | 49 | 0.53 (0.39-0.70) |
| Aunt | 8,709 | 0.57 (0.55-0.58) | 164 | 0.57 (0.49-0.66) | 25 | 0.52 (0.34-0.77) |
| Uncle | 8,772 | 0.60 (0.58-0.61) | 169 | 0.62 (0.53-0.73) | 24 | 0.54 (0.35-0.81) |
| **Alzheimer’s disease** | | | | | | |
| Parents | 13,750 | 0.37 (0.36-0.37) | 312 | 0.45 (0.40-0.50) | 57 | 0.49 (0.37-0.64) |
| Mother | 4,742 | 0.29 (0.28-0.29) | 117 | 0.39 (0.32-0.47) | 16 | 0.33 (0.19-0.53) |
| Father | 9,008 | 0.43 (0.43-0.44) | 195 | 0.49 (0.43-0.57) | 41 | 0.62 (0.44-0.83) |
| Grandparents | 840,274 | 3.36 (3.36-3.37) | 17,666 | 3.20 (3.16-3.25) | 2,757 | 3.24 (3.12-3.37) |
| Grandmother | 471,035 | 3.67 (3.66-3.68) | 9,537 | 3.38 (3.32-3.45) | 1,524 | 3.51 (3.34-3.69) |
| Grandfather | 369,239 | 3.04 (3.03-3.05) | 8,129 | 3.02 (2.95-3.08) | 1,233 | 2.96 (2.80-3.13) |
| Uncles/aunts | 13,992 | 0.46 (0.46-0.47) | 266 | 0.48 (0.42-0.54) | 40 | 0.43 (0.31-0.59) |
| Aunt | 7,365 | 0.48 (0.47-0.49) | 142 | 0.49 (0.42-0.58) | 19 | 0.39 (0.24-0.62) |
| Uncle | 6,627 | 0.45 (0.44-0.46) | 124 | 0.46 (0.38-0.55) | 21 | 0.48 (0.30-0.73) |
| **Other dementia** | | | | | | |
| Parents | 6,870 | 0.18 (0.18-0.19) | 157 | 0.23 (0.19-0.26) | 32 | 0.28 (0.19-0.39) |
| Mother | 1,658 | 0.10 (0.10-0.10) | 41 | 0.14 (0.10-0.19) | 8 | 0.16 (0.07-0.32) |
| Father | 5,212 | 0.25 (0.24-0.26) | 116 | 0.29 (0.24-0.35) | 24 | 0.36 (0.23-0.53) |
| Grandparents | 292,232 | 1.15 (1.15-1.16) | 6,431 | 1.15 (1.12-1.18) | 1,021 | 1.19 (1.11-1.26) |
| Grandmother | 138,015 | 1.06 (1.05-1.06) | 2,956 | 1.03 (1.00-1.07) | 474 | 1.07 (0.98-1.18) |
| Grandfather | 154,217 | 1.26 (1.25-1.26) | 3,475 | 1.28 (1.23-1.32) | 547 | 1.30 (1.19-1.41) |
| Uncles/aunts | 6,103 | 0.20 (0.20-0.21) | 128 | 0.23 (0.19-0.27) | 13 | 0.14 (0.07-0.24) |
| Aunt | 2,469 | 0.16 (0.15-0.17) | 45 | 0.16 (0.11-0.21) | 6 | 0.12 (0.05-0.27) |
| Uncle | 3,634 | 0.25 (0.24-0.25) | 83 | 0.31 (0.24-0.38) | 7 | 0.16 (0.06-0.33) |

CI, confidence interval; HR, hazard ratio; ID, intelligence disability.

**Supplementary Table S6** Associations between autism and any dementia/Alzheimer's disease/other dementia across generations stratified by intellectual disability in index persons

| **Relative cohorts** | **Autistic index person without intellectual disability** | | **Autistic index person with intellectual disability** | |
| --- | --- | --- | --- | --- |
|  | **Crude HR**  **(95% CI)** | **Adjusted HR**  **(95% CI)**** | **Crude HR**  **(95% CI)** | **Adjusted HR**  **(95% CI)*** |
| **Any dementia** |  |  |  |  |
| Parents | 1.33 (1.20-1.47) | 1.35 (1.22-1.49) | 1.44 (1.15-1.81) | 1.44 (1.15-1.80) |
| Mother | 1.44 (1.22-1.71) | 1.53 (1.29-1.81) | 1.33 (0.87-2.05) | 1.42 (0.92-2.18) |
| Father | 1.26 (1.12-1.43) | 1.27 (1.13-1.44) | 1.47 (1.13-1.91) | 1.44 (1.10-1.87) |
| Grandparents | 1.06 (1.04-1.07) | 1.09 (1.07-1.10) | 1.06 (1.03-1.10) | 1.08 (1.05-1.12) |
| Grandmother | 1.05 (1.03-1.07) | 1.08 (1.06-1.10) | 1.06 (1.01-1.11) | 1.09 (1.04-1.14) |
| Grandfather | 1.07 (1.05-1.09) | 1.09 (1.07-1.11) | 1.07 (1.02-1.12) | 1.08 (1.03-1.14) |
| Uncles/aunts | 1.13 (1.01-1.26) | 1.14 (1.03-1.28) | 0.97 (0.74-1.29) | 0.98 (0.74-1.30) |
| Aunt | 1.11 (0.95-1.29) | 1.12 (0.96-1.31) | 0.99 (0.67-1.47) | 1.01 (0.68-1.49) |
| Uncle | 1.15 (0.99-1.34) | 1.17 (1.00-1.36) | 0.96 (0.64-1.44) | 0.95 (0.63-1.42) |
| **Alzheimer’s disease** |  |  |  |  |
| Parents | 1.32 (1.18-1.47) | 1.34 (1.20-1.50) | 1.35 (1.04-1.76) | 1.35 (1.03-1.75) |
| Mother | 1.50 (1.25-1.80) | 1.58 (1.32-1.90) | 1.23 (0.75-2.00) | 1.30 (0.80-2.12) |
| Father | 1.22 (1.06-1.40) | 1.23 (1.06-1.41) | 1.39 (1.02-1.89) | 1.36 (1.00-1.85) |
| Grandparents | 1.06 (1.04-1.07) | 1.09 (1.07-1.10) | 1.05 (1.01-1.09) | 1.07 (1.03-1.11) |
| Grandmother | 1.05 (1.02-1.07) | 1.08 (1.06-1.10) | 1.06 (1.01-1.11) | 1.08 (1.03-1.14) |
| Grandfather | 1.07 (1.05-1.10) | 1.09 (1.07-1.12) | 1.04 (0.98-1.10) | 1.05 (1.00-1.11) |
| Uncles/aunts | 1.13 (1.00-1.28) | 1.14 (1.01-1.29) | 1.00 (0.73-1.36) | 1.00 (0.73-1.36) |
| Aunt | 1.14 (0.96-1.34) | 1.15 (0.98-1.36) | 0.89 (0.57-1.40) | 0.91 (0.58-1.43) |
| Uncle | 1.12 (0.93-1.34) | 1.13 (0.95-1.35) | 1.12 (0.73-1.71) | 1.09 (0.71-1.68) |
| **Other dementia** |  |  |  |  |
| Parents | 1.33 (1.14-1.56) | 1.34 (1.14-1.57) | 1.52 (1.07-2.15) | 1.48 (1.05-2.10) |
| Mother | 1.49 (1.09-2.03) | 1.60 (1.17-2.18) | 1.74 (0.87-3.50) | 1.88 (0.94-3.77) |
| Father | 1.26 (1.05-1.51) | 1.26 (1.05-1.52) | 1.42 (0.95-2.12) | 1.38 (0.92-2.06) |
| Grandparents | 1.09 (1.06-1.11) | 1.10 (1.07-1.13) | 1.10 (1.03-1.17) | 1.11 (1.04-1.18) |
| Grandmother | 1.09 (1.05-1.13) | 1.12 (1.08-1.16) | 1.11 (1.02-1.22) | 1.13 (1.04-1.24) |
| Grandfather | 1.08 (1.04-1.12) | 1.08 (1.05-1.12) | 1.09 (1.00-1.18) | 1.09 (1.00-1.19) |
| Uncles/aunts | 1.25 (1.05-1.48) | 1.27 (1.07-1.52) | 0.74 (0.43-1.28) | 0.75 (0.43-1.29) |
| Aunt | 1.07 (0.80-1.44) | 1.08 (0.81-1.45) | 0.84 (0.38-1.87) | 0.85 (0.38-1.90) |
| Uncle | 1.37 (1.10-1.70) | 1.41 (1.13-1.75) | 0.68 (0.32-1.42) | 0.68 (0.32-1.42) |

CI, confidence interval; HR, hazard ratio; ID, intelligence disability.

* Adjusted for index sex, index birth year categories, and relative birth year categories

**Supplementary Table S7** Sensitivity analysis: associations between autism and any dementia/Alzheimer's disease/other dementia across generations: refining diagnosis and death timing in dementia

| **Relative cohorts** | **Autistic index person** | | **Nonautistic index person** | | **Crude HR**  **(95% CI)** | **Adjusted HR**  **(95% CI)**** |
| --- | --- | --- | --- | --- | --- | --- |
|  | **No. of events** | **Incidence rate** | **No. of events** | **Incidence rate** |  |  |
| **Any dementia** | | | | | | |
| Parents | 501 | 0.62 (0.57-0.67) | 18,100 | 0.48 (0.48-0.49) | 1.36 (1.24-1.48) | 1.36 (1.24-1.48) |
| Mother | 165 | 0.48 (0.41-0.55) | 5,934 | 0.36 (0.35-0.37) | 1.49 (1.27-1.74) | 1.50 (1.29-1.75) |
| Father | 336 | 0.73 (0.65-0.81) | 12,166 | 0.59 (0.58-0.60) | 1.30 (1.16-1.44) | 1.29 (1.16-1.44) |
| Grandparents | 23,856 | 3.71 (3.67-3.76) | 975,791 | 3.87 (3.86-3.88) | 1.10 (1.09-1.12) | 1.10 (1.08-1.11) |
| Grandmother | 12,573 | 3.83 (3.76-3.89) | 533,335 | 4.11 (4.10-4.12) | 1.10 (1.08-1.12) | 1.10 (1.08-1.12) |
| Grandfather | 11,283 | 3.60 (3.53-3.66) | 442,456 | 3.61 (3.60-3.63) | 1.11 (1.09-1.13) | 1.10 (1.08-1.12) |
| Uncles/aunts | 388 | 0.60 (0.54-0.66) | 17,797 | 0.59 (0.58-0.60) | 1.12 (1.01-1.24) | 1.11 (1.01-1.23) |
| Aunt | 194 | 0.58 (0.50-0.66) | 8,896 | 0.58 (0.56-0.59) | 1.11 (0.96-1.28) | 1.10 (0.95-1.27) |
| Uncle | 194 | 0.62 (0.53-0.71) | 8,901 | 0.60 (0.59-0.62) | 1.13 (0.98-1.31) | 1.13 (0.98-1.30) |
| **Alzheimer’s disease** | | | | | | |
| Parents | 381 | 0.47 (0.42-0.52) | 14,040 | 0.38 (0.37-0.38) | 1.33 (1.20-1.47) | 1.33 (1.20-1.47) |
| Mother | 139 | 0.40 (0.34-0.47) | 4,921 | 0.30 (0.29-0.30) | 1.52 (1.28-1.79) | 1.53 (1.29-1.81) |
| Father | 242 | 0.52 (0.46-0.59) | 9,119 | 0.44 (0.43-0.45) | 1.24 (1.09-1.41) | 1.24 (1.09-1.41) |
| Grandparents | 20,450 | 3.18 (3.13-3.22) | 840,946 | 3.33 (3.32-3.33) | 1.10 (1.09-1.12) | 1.09 (1.08-1.11) |
| Grandmother | 11,074 | 3.36 (3.30-3.43) | 471,359 | 3.62 (3.61-3.63) | 1.10 (1.08-1.12) | 1.09 (1.07-1.12) |
| Grandfather | 9376 | 2.98 (2.92-3.04) | 369,587 | 3.01 (3.00-3.02) | 1.11 (1.09-1.13) | 1.10 (1.07-1.12) |
| Uncles/aunts | 311 | 0.48 (0.43-0.53) | 14,202 | 0.47 (0.46-0.48) | 1.13 (1.01-1.26) | 1.12 (1.00-1.26) |
| Aunt | 165 | 0.49 (0.42-0.57) | 7,504 | 0.49 (0.48-0.50) | 1.12 (0.96-1.31) | 1.12 (0.96-1.30) |
| Uncle | 146 | 0.46 (0.39-0.54) | 6,698 | 0.45 (0.44-0.46) | 1.14 (0.96-1.34) | 1.13 (0.96-1.33) |
| **Other dementia** | | | | | | |
| Parents | 198 | 0.24 (0.21-0.28) | 7,095 | 0.19 (0.19-0.19) | 1.37 (1.18-1.57) | 1.35 (1.17-1.56) |
| Mother | 51 | 0.15 (0.11-0.19) | 1,743 | 0.10 (0.10-0.11) | 1.55 (1.18-2.05) | 1.59 (1.20-2.10) |
| Father | 147 | 0.32 (0.27-0.37) | 5,352 | 0.26 (0.25-0.26) | 1.29 (1.09-1.52) | 1.28 (1.08-1.51) |
| Grandparents | 7,465 | 1.15 (1.13-1.18) | 292,633 | 1.15 (1.15-1.16) | 1.14 (1.11-1.16) | 1.11 (1.09-1.14) |
| Grandmother | 3,440 | 1.04 (1.00-1.07) | 138,204 | 1.05 (1.05-1.06) | 1.15 (1.11-1.19) | 1.14 (1.10-1.18) |
| Grandfather | 4,025 | 1.28 (1.24-1.32) | 154,429 | 1.25 (1.25-1.26) | 1.12 (1.08-1.15) | 1.09 (1.06-1.13) |
| Uncles/aunts | 143 | 0.22 (0.18-0.26) | 6,262 | 0.21 (0.20-0.21) | 1.18 (1.00-1.39) | 1.17 (1.00-1.39) |
| Aunt | 52 | 0.15 (0.12-0.20) | 2,537 | 0.16 (0.16-0.17) | 1.04 (0.79-1.37) | 1.03 (0.78-1.35) |
| Uncle | 91 | 0.29 (0.23-0.35) | 3,725 | 0.25 (0.24-0.26) | 1.27 (1.03-1.57) | 1.28 (1.04-1.58) |

CI, confidence interval; HR, hazard ratio

* Adjusted for index sex, index birth year categories, and relative birth year categories

**Supplementary Table S8** Sensitivity analysis: associations between autism and any dementia/Alzheimer's disease/other dementia across generations after adding Alzheimer's disease medication to define outcomes

| **Relative cohorts** | **Autistic index person** | | **Nonautistic index person** | | **Crude HR**  **(95% CI)** | **Adjusted HR**  **(95% CI)*** |
| --- | --- | --- | --- | --- | --- | --- |
|  | **No. of events** | **Incidence rate** | **No. of events** | **Incidence rate** |  |  |
| **Any dementia** | | | | | | |
| Parents | 564 | 0.70 (0.64-0.76) | 21,239 | 0.57 (0.56-0.58) | 1.31 (1.20-1.42) | 1.32 (1.22-1.44) |
| Mother | 189 | 0.55 (0.47-0.63) | 6,937 | 0.42 (0.41-0.43) | 1.42 (1.23-1.64) | 1.48 (1.28-1.72) |
| Father | 375 | 0.81 (0.73-0.90) | 14,302 | 0.69 (0.68-0.70) | 1.25 (1.13-1.38) | 1.25 (1.13-1.39) |
| Grandparents | 26,147 | 4.13 (4.08-4.18) | 1,066,928 | 4.29 (4.29-4.30) | 1.06 (1.05-1.08) | 1.08 (1.07-1.09) |
| Grandmother | 13,907 | 4.30 (4.22-4.37) | 587,068 | 4.60 (4.58-4.61) | 1.06 (1.04-1.08) | 1.08 (1.06-1.10) |
| Grandfather | 12,240 | 3.95 (3.88-4.03) | 479,860 | 3.97 (3.96-3.99) | 1.07 (1.05-1.09) | 1.08 (1.06-1.10) |
| Uncles/aunts | 472 | 0.73 (0.66-0.79) | 21,273 | 0.71 (0.70-0.72) | 1.13 (1.03-1.24) | 1.14 (1.04-1.25) |
| Aunt | 236 | 0.70 (0.62-0.80) | 10,683 | 0.69 (0.68-0.71) | 1.11 (0.98-1.27) | 1.12 (0.99-1.28) |
| Uncle | 236 | 0.75 (0.66-0.85) | 10,590 | 0.72 (0.70-0.73) | 1.14 (1.00-1.30) | 1.16 (1.02-1.32) |
| **Alzheimer’s disease** | | | | | | |
| Parents | 489 | 0.60 (0.55-0.66) | 18,634 | 0.50 (0.49-0.51) | 1.29 (1.18-1.42) | 1.31 (1.20-1.43) |
| Mother | 176 | 0.51 (0.44-0.59) | 6,264 | 0.38 (0.37-0.39) | 1.47 (1.27-1.71) | 1.53 (1.32-1.78) |
| Father | 313 | 0.68 (0.60-0.76) | 12,370 | 0.60 (0.59-0.61) | 1.20 (1.08-1.35) | 1.21 (1.08-1.35) |
| Grandparents | 23,475 | 3.69 (3.65-3.74) | 960,972 | 3.85 (3.85-3.86) | 1.06 (1.05-1.08) | 1.07 (1.06-1.09) |
| Grandmother | 12,744 | 3.92 (3.86-3.99) | 538,133 | 4.20 (4.19-4.21) | 1.06 (1.04-1.08) | 1.08 (1.06-1.10) |
| Grandfather | 10,731 | 3.45 (3.39-3.52) | 422,839 | 3.49 (3.48-3.50) | 1.07 (1.05-1.09) | 1.07 (1.05-1.09) |
| Uncles/aunts | 428 | 0.66 (0.60-0.72) | 19,059 | 0.63 (0.62-0.64) | 1.15 (1.04-1.26) | 1.15 (1.05-1.27) |
| Aunt | 217 | 0.65 (0.56-0.74) | 9,777 | 0.63 (0.62-0.65) | 1.12 (0.98-1.28) | 1.13 (0.99-1.29) |
| Uncle | 211 | 0.67 (0.58-0.77) | 9,282 | 0.63 (0.62-0.64) | 1.17 (1.02-1.34) | 1.18 (1.02-1.35) |

CI, confidence interval; HR, hazard ratio

* Adjusted for index sex, index birth year categories, and relative birth year categories

**Supplementary Table S9** Sensitivity analysis: associations between autism and any dementia/Alzheimer's disease/other dementia across generations among index individuals born between 1980 and 2005

| **Relative cohorts** | **Autistic index person** | | **Nonautistic index person** | | **Crude HR**  **(95% CI)** | **Adjusted HR**  **(95% CI)*** |
| --- | --- | --- | --- | --- | --- | --- |
|  | **No. of events** | **Incidence rate** | **No. of events** | **Incidence rate** |  |  |
| **Any dementia** | | | | | | |
| Parents | 480 | 0.61 (0.55-0.66) | 17,610 | 0.48 (0.47-0.49) | 1.35 (1.23-1.48) | 1.37 (1.25-1.50) |
| Mother | 157 | 0.46 (0.39-0.53) | 5,705 | 0.35 (0.34-0.36) | 1.43 (1.22-1.68) | 1.51 (1.29-1.77) |
| Father | 323 | 0.72 (0.64-0.80) | 11,905 | 0.59 (0.58-0.60) | 1.30 (1.16-1.45) | 1.30 (1.16-1.45) |
| Grandparents | 22,105 | 4.13 (4.07-4.18) | 909,336 | 4.44 (4.43-4.45) | 1.05 (1.04-1.07) | 1.08 (1.06-1.09) |
| Grandmother | 11,828 | 4.28 (4.21-4.36) | 505,496 | 4.75 (4.73-4.76) | 1.04 (1.03-1.06) | 1.08 (1.06-1.10) |
| Grandfather | 10,277 | 3.96 (3.88-4.03) | 403,840 | 4.11 (4.10-4.12) | 1.06 (1.04-1.08) | 1.08 (1.06-1.10) |
| Uncles/aunts | 377 | 0.60 (0.54-0.66) | 17,354 | 0.59 (0.58-0.60) | 1.11 (1.00-1.22) | 1.12 (1.01-1.24) |
| Aunt | 186 | 0.57 (0.49-0.66) | 8,646 | 0.58 (0.56-0.59) | 1.09 (0.94-1.26) | 1.10 (0.95-1.27) |
| Uncle | 191 | 0.63 (0.54-0.72) | 8,708 | 0.61 (0.59-0.62) | 1.12 (0.97-1.30) | 1.13 (0.98-1.31) |
| **Alzheimer’s disease** | | | | | | |
| Parents | 369 | 0.47 (0.42-0.52) | 13,710 | 0.37 (0.37-0.38) | 1.33 (1.20-1.48) | 1.35 (1.22-1.49) |
| Mother | 133 | 0.39 (0.32-0.46) | 4,740 | 0.29 (0.28-0.30) | 1.46 (1.23-1.74) | 1.54 (1.30-1.83) |
| Father | 236 | 0.52 (0.46-0.60) | 8,970 | 0.44 (0.43-0.45) | 1.26 (1.10-1.43) | 1.25 (1.10-1.43) |
| Grandparents | 18,998 | 3.53 (3.48-3.58) | 786,478 | 3.83 (3.82-3.83) | 1.05 (1.03-1.06) | 1.08 (1.06-1.09) |
| Grandmother | 10,436 | 3.77 (3.69-3.84) | 447,492 | 4.19 (4.17-4.20) | 1.04 (1.02-1.06) | 1.08 (1.06-1.10) |
| Grandfather | 8,562 | 3.28 (3.21-3.35) | 338,986 | 3.44 (3.42-3.45) | 1.06 (1.04-1.08) | 1.08 (1.05-1.10) |
| Uncles/aunts | 304 | 0.48 (0.43-0.54) | 13,894 | 0.47 (0.46-0.48) | 1.12 (1.00-1.25) | 1.13 (1.00-1.26) |
| Aunt | 159 | 0.49 (0.42-0.57) | 7,312 | 0.49 (0.48-0.50) | 1.10 (0.94-1.29) | 1.12 (0.95-1.31) |
| Uncle | 145 | 0.47 (0.40-0.56) | 6,582 | 0.46 (0.45-0.47) | 1.13 (0.96-1.33) | 1.14 (0.96-1.34) |
| **Other dementia** | | | | | | |
| Parents | 187 | 0.24 (0.20-0.27) | 6,849 | 0.19 (0.18-0.19) | 1.36 (1.17-1.57) | 1.35 (1.17-1.57) |
| Mother | 49 | 0.14 (0.11-0.19) | 1,658 | 0.10 (0.10-0.11) | 1.53 (1.15-2.03) | 1.64 (1.23-2.17) |
| Father | 138 | 0.31 (0.26-0.36) | 5,191 | 0.26 (0.25-0.26) | 1.28 (1.08-1.51) | 1.27 (1.07-1.50) |
| Grandparents | 6,843 | 1.25 (1.22-1.28) | 269,222 | 1.29 (1.28-1.29) | 1.08 (1.06-1.11) | 1.10 (1.07-1.12) |
| Grandmother | 3,222 | 1.14 (1.10-1.18) | 130,409 | 1.20 (1.19-1.20) | 1.09 (1.05-1.13) | 1.12 (1.08-1.16) |
| Grandfather | 3,621 | 1.37 (1.33-1.42) | 138,813 | 1.39 (1.38-1.40) | 1.07 (1.04-1.11) | 1.07 (1.04-1.11) |
| Uncles/aunts | 138 | 0.22 (0.18-0.26) | 6,066 | 0.21 (0.20-0.21) | 1.16 (0.98-1.37) | 1.18 (1.00-1.40) |
| Aunt | 50 | 0.15 (0.11-0.20) | 2,456 | 0.16 (0.16-0.17) | 1.03 (0.78-1.36) | 1.04 (0.78-1.37) |
| Uncle | 88 | 0.29 (0.23-0.35) | 3,610 | 0.25 (0.24-0.26) | 1.25 (1.01-1.55) | 1.28 (1.04-1.59) |

CI, confidence interval; HR, hazard ratio

* Adjusted for index sex, index birth year categories, and relative birth year categories

Note: index-parents pairs: 3,470,662; index-grandparents pairs 7,775,448; index-aunt/uncle pairs: 2,567,713

**Supplementary Table S10** Association between number of autism in children/grandchildren and risk of dementia.

|  | Number of individuals | Number of any dementia | Adjusted HR |
| --- | --- | --- | --- |
| Number of autistic children |  |  |  |
| 0 | 6,119,063 | 147,583 | Ref |
| 1 | 72,245 | 1,656 | 1.13 (1.07-1.18) |
| >= 2 | 14,925 | 67 | 1.39 (1.09-1.77) |
| Number of autistic grandchildren |  |  |  |
| 0 | 8,239,899 | 138,028 | Ref |
| 1 | 852,957 | 9,641 | 1.07 (1.05-1.09) |
| >= 2 | 181,451 | 1,637 | 1.18 (1.12-1.24) |

HRs were adjusted for sex, birth year category, number of children or grandchildren.
